# Symptom reporting and quality of life in the Estonian Postmenopausal Hormone Therapy Trial

**DOI:** 10.1186/1472-6874-8-5

**Published:** 2008-03-26

**Authors:** Piret Veerus, Krista Fischer, Sirpa-Liisa Hovi, Helle Karro, Mati Rahu, Elina Hemminki

**Affiliations:** 1 Department of Epidemiology and Biostatistics, National Institute for Health Development, Hiiu 42, 11619 Tallinn, Estonia; 2 MRC Biostatistics Unit, Institute of Public Health, Robinson Way, Cambridge CB2 0SR, UK; 3 Department of Health and Social Services, National Research and Development Centre for Welfare and Health, STAKES, PO Box 220, FI-00531 Helsinki, Finland; 4 Tartu University Women's Clinic, Lossi 36, 51003 Tartu, Estonia

## Abstract

**Background:**

The aim of the study was to determine the effect of postmenopausal hormone therapy on women's symptom reporting and quality of life in a randomized trial.

**Methods:**

1823 women participated in the Estonian Postmenopausal Hormone Therapy (EPHT) Trial between 1999 and 2004. Women were randomized to open-label continuous combined hormone therapy or no treatment, or to blind hormone therapy or placebo. The average follow-up period was 3.6 years. Prevalence of symptoms and quality of life according to EQ-5D were assessed by annually mailed questionnaires.

**Results:**

In the hormone therapy arms, less women reported hot flushes (OR 0.20; 95% CI: 0.14–0.28), sweating (OR 0.56; 95% CI: 0.44–0.72), and sleeping problems (OR 0.66; 95% CI: 0.52–0.84), but more women reported episodes of vaginal bleeding (OR 19.65; 95% CI: 12.15–31.79). There was no difference between the trial arms in the prevalence of other symptoms over time. Quality of life did not depend on hormone therapy use.

**Conclusion:**

Postmenopausal hormone therapy decreased vasomotor symptoms and sleeping problems, but increased episodes of vaginal bleeding, and had no effect on quality of life.

**Trial registration number:**

ISRCTN35338757

## Background

The effect of menopausal transition on women's lives is complex and includes changes in physical health, psychosomatic domains, and personal life [1]. Health-related quality of life may be severely compromised in women with vasomotor symptoms [2]. Up to 40% of women in Sweden experience vasomotor symptoms until the age of 64 years [3]. Postmenopausal hormone therapy has for a long time been the recommended first choice to alleviate hot flushes and night sweats [4-7]. Hormone therapy may have side effects such as vaginal bleeding, breast tenderness, migraine headaches, mood alterations, and abdominal bloating that may affect quality of life [8]. Besides vasomotor symptoms, sleep and sexual problems are related to postmenopausal status [9,10].

Population-based studies indicate that hormone therapy improves vasomotor symptoms and sexual problems [11], but not quality of life [12]. In the Heart and Estrogen/Progestin Replacement Study (HERS), women with hot flushes had improvements in emotional measures of quality of life [13]. In the Women's Health Initiative (WHI) Trial, combined hormone therapy relieved some menopausal symptoms, but contributed to side effects [14], and no clinically meaningful effect on health-related quality of life was found [15,16].

The Estonian Postmenopausal Hormone Therapy (EPHT) Trial was a long-term preventive trial of hormone therapy among healthy postmenopausal women. The EPHT Trial consisted of two sub-trials: one blind and one non-blind. Women were randomly allocated to combined continuous hormone therapy or non-treatment, or to blind hormone therapy or placebo. Their baseline health indicators showed better health than those of the participants in the WHI or HERS [17]. At baseline, women in the EPHT Trial were aged on average 58 and were 8 years postmenopausal compared to an average age of 63 years (13 years postmenopausal) in the WHI Trial [14] and 67 years (18 years postmenopausal) in the HERS Trial [13,18].

Besides studying the effect of postmenopausal hormone therapy on the risk of cancer, cardiovascular diseases and bone fractures [17] and the related use of health services [19], the other major aims of the EPHT Trial were to analyze the effect of hormone therapy on symptom reporting and health-related quality of life.

## Methods

### Participants

**Figure 1:**
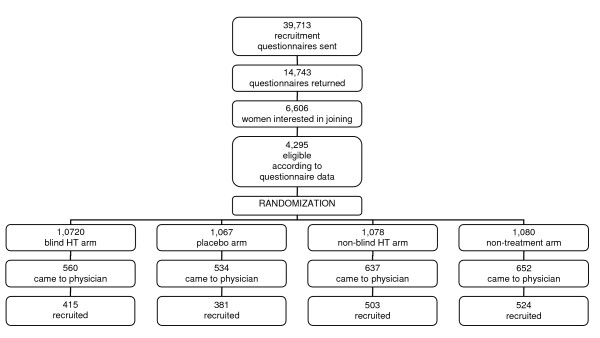
Flow chart of the EPHT Trial.

Postmenopausal women aged 50 to 64 and living in Tallinn (the capital of Estonia), Tartu, and in two counties surrounding these towns were asked to participate in a postmenopausal hormone therapy trial. Potentially eligible women were randomized into four trial arms: 1) blind hormone therapy arm, 2) blind placebo arm, 3) non-blind hormone therapy arm, 4) non-treatment arm. Randomization occurred before mailing the invitation to the recruitment visit in order to study the impact of blinding on recruitment. Randomized women were mailed an invitation to the recruitment visit, indicating whether they had been randomized to the blind or the non-blind sub-trial (Figure 1). The non-blind sub-trial was designed to study the impact of hormone therapy on health service utilization.

More women came to the recruitment visit in the non-blind sub-trial, and more women signed the informed consent during the recruitment visit in the non-blind sub-trial. This resulted in 503 women recruited in the non-blind hormone therapy arm, 524 in the non-treatment arm, 415 in the blind hormone therapy arm, and 381 in the blind placebo arm. Altogether 1823 participants were recruited at three clinical centers between January 1999 and December 2001. Details of the trial design, randomization, eligibility criteria, recruitment, and clinical follow-up of participants are described elsewhere [17]. Trial treatment was stopped by May 2004. The mean follow-up period for trial participants was 3.6 years. All participants gave written informed consent. The trial design was approved by the Tallinn Medical Research Ethics Committee.

At baseline, the mean age of participants was 58.2 years (SD 4.0 yrs) and they were on average 8.0 years (SD 4.0) postmenopausal. Of the recruited women, 15% were current smokers, 13% had been treated for hypertension, 1.9% had a history of stenocardia, 0.3% a history of myocardial infarction, and 1.6% a history of diabetes. The mean body mass index of the trial participants at baseline was 27.0 kg/m^2^. At the time of recruitment, 62% were married and 69% still working, with 33% of participants having a higher education. The mean parity of the participants was 1.8. At the time of joining the trial, 52% of participating women were living in the capital, 17% around it, 21% in a small town, and 10% in the countryside. There were no significant differences in the background characteristics of women in different trial arms over time.

Participants in the hormone therapy arms received 0.625 mg of combined estrogens plus 2.5 mg of medroxyprogesterone acetate daily orally or a matched placebo in the placebo arm or no treatment in the control arm. The 251 participants who were within three years of their last menstrual period received 0.625 mg of combined estrogens plus 5.0 mg of medroxyprogesterone acetate daily orally or matched placebo or no treatment. A general algorithm was followed for the management of bleeding depending on the severity and duration of bleeding.

At the end of the first trial year, 83% of the participants took over 20% of their assigned trial medication (82% in the placebo arm), with 57% of participants for the second year (54% in the placebo arm), 64% of participants for the third year (54% in the placebo arm), and 63% of participants for the whole trial period in the hormone therapy arms using more than 20% of allocated trial treatment (the mean proportion of adherent women in the placebo arm for the whole trial period was 59%). Throughout the trial, some 90% of women in the non-treatment arm did not start hormone therapy. In the placebo arm, 5% of women started prescribed hormone therapy. Reasons for non-adherence have been reported separately [20].

In the non-blind sub-trial, participants and trial staff were aware of the treatment allocation. In the blind sub-trial, participants and physicians were blinded as to the composition of trial treatment. These women were informed about the composition of their drug within one month of the closure visit.

### Data collection

Prevalence of symptoms was assessed by mailed questionnaires before recruitment, at the end of each trial year, and at the end of the trial. Quality of life was assessed by calculating EuroQoL (EQ-5D) scores [21] at the end of the second trial year and also at the time of stopping the trial.

All annual questionnaires included questions about the prevalence of 17 symptoms in the previous two weeks (dizzy spells, chronic fatigue, diarrhea or constipation, irritability, persistent cough, depression, backaches, upset stomach, headaches, sweating, aches/stiffness in the joints, shortness of breath, hot flushes, sore throat, trouble sleeping, loss of appetite, water retention) to evaluate the physical health and psychological status of trial participants. The same questions were asked of all participants before recruitment. All annual questionnaires contained questions about the presence, number and severity of bleeding episodes in the previous 12 months. The first and the final annual questionnaire included questions about painful intercourse in the previous 12 months.

The second annual and the final questionnaires contained EQ-5D [21]. EQ-5D is a standardized instrument for measuring health outcomes. It contains five questions asking whether the respondent has problems with mobility, self-care, usual activities, pain/discomfort and anxiety/depression with three possible responses available (no problems, moderate problems, severe problems). EQ-5D is designed for self-completion and provides a single index score for health status. The lowest possible value for the EQ-5D index is 0 and the highest possible value is 1.

All participants filled in the recruitment questionnaire. The response rate was 75% for the first annual survey, 69% for the second annual survey, and 81% for the final survey mailed at the end of the trial and followed by one reminder. On average, the final survey was filled in 3.6 years after the recruitment. There were no significant differences in the annual response rates between the trial arms (Table 1).

**Table 1:** Number and proportion of women responding to annual questionnaires by trial arm in the EPHT Trial, 1999–2004

Trial arm	Recruitment N (%)	1^st ^year N (%)	2^nd ^year N (%)	Final N (%)
Blind HT	415 (100)	315 (75.9)	295 (71.1)	329 (79.3)
Blind placebo	381 (100)	278 (73.0)	254 (66.7)	308 (80.8)
Non-blind HT	503 (100)	371 (73.8)	340 (67.6)	405 (80.5)
Non-treatment	524 (100)	395 (75.4)	374 (71.4)	445 (84.9)
Total	1823 (100)	1359 (74.6)	1263 (69.3)	1487 (81.4)

### Data analysis

We used R version 2.5.0 [22] to analyze the data on an intention-to-treat basis. Numbers of women reporting vaginal bleeding and number and severity of bleeding episodes were compared in different trial arms at the end of the second trial year and at the end of the trial. The proportion of women reporting painful intercourse in different trial arms was compared at the end of the first trial year and at the end of the trial.

For the set of binary indicators on symptom prevalence, longitudinal data analysis as mixed effects logistic regression with random subject-specific intercepts, using a penalized quasi-likelihood method and assuming a first-order autoregressive dependence structure within subjects was performed to test if the prevalence of different symptoms over time depended on hormone treatment. The model was adjusted for the presence of these symptoms at baseline, for time, participant's age and being randomized to a blind sub-trial or not. In the fitting of models, the blind and the non-blind hormone therapy arms were combined as well as the placebo and non-treatment arms to increase power.

EQ-5D scores were compared in all trial arms at the end of the second trial year and at the end of the trial. The EQ-5D score was calculated using the simple formula based method [23]. Longitudinal data analysis as general linear mixed effects modeling using restricted maximum likelihood and assuming exponential spatial correlation between measurements (strength of which depending on the length of the time interval between measurements) was used to analyze the effect of age, treatment, time and blinding on the quality of life.

## Results

Symptoms reported most often by all participants at recruitment were aches or stiffness in joints (56%), chronic fatigue (53%), sweating (47%), hot flushes (45%), and backache (40%). There were no baseline differences in the prevalence of symptoms in different arms except for sweating, which was reported more often by women in the hormone therapy arm (50%) than in the non-treatment arm (44%) of the non-blind sub-trial (Table 2).

**Table 2:** Proportion of women reporting different symptoms in each arm of the EPHT Trial over time* and the effect of hormone therapy on symptom reporting

Symptom	Non- blind HT (%)	Non- treatment (%)	Blind HT (%)	Placebo (%)	OR (95% CI)
Hot flushes					
recruitment	43.4	41.1	46.2	48.4	0.20 (0.14–0.28)
2^nd ^year	10.8	22.9	9.1	26.2	
final	14.6	23.6	13.1	22.5	
Sweating					
recruitment	50.0	43.5	46.2	47.2	0.56 (0.44–0.72)
2^nd ^year	23.9	32.4	22.9	32.8	
final	26.4	31.8	24.0	31.4	
Trouble sleeping					
recruitment	31.4	30.3	30.2	34.3	0.66 (0.52–0.84)
2^nd ^year	25.6	31.1	24.9	32.0	
final	34.1	36.2	31.3	33.3	
Depression					
recruitment	27.1	27.2	23.4	21.0	0.81 (0.60–1.08)
2^nd ^year	19.9	21.1	19.2	22.8	
final	21.6	23.6	18.9	19.3	
Anxiety					
recruitment	34.4	36.1	34.6	33.2	0.93 (0.73–1.19)
2^nd ^year	27.1	25.7	28.3	27.8	
final	27.3	29.5	25.2	25.2	
Backache					
recruitment	40.2	40.0	42.1	36.0	1.15 (0.96–1.38)
2^nd ^year	38.7	37.0	35.7	37.3	
final	45.4	37.2	39.5	35.6	
Stiffness/aches in joints					
recruitment	57.5	54.5	56.3	54.2	0.97 (0.82–1.15)
2^nd ^year	50.3	56.4	50.8	57.0	
final	57.5	56.5	54.4	56.5	
Chronic fatigue					
recruitment	55.5	53.9	51.7	49.0	1.11 (0.90–1.35)
2^nd ^year	52.8	51.6	47.5	49.4	
final	55.3	49.5	47.7	49.3	
Headache					
recruitment	39.1	33.3	35.1	38.8	1.08 (0.76–1.54)
2^nd ^year	34.1	30.1	33.3	32.4	
final	35.7	30.9	35.3	33.3	
Dizzy spells					
recruitment	26.6	23.3	21.1	24.7	0.97 (0.74–1.28)
2^nd ^year	18.7	22.3	21.2	19.6	
final	19.4	23.0	19.5	16.7	
Diarrhoea or constipation					
recruitment	26.4	26.6	21.2	21.7	0.99 (0.75–1.30)
2^nd ^year	24.8	20.2	18.6	19.9	
final	24.8	24.3	24.3	24.5	
Upset stomach					
recruitment	12.3	13.3	11.0	7.6	0.92 (0.66–1.29)
2^nd ^year'	11.6	10.1	9.1	10.9	
final	9.4	8.3	10.6	8.2	
Shortness of breath					
recruitment	20.0	14.9	18.5	13.9	0.97 (0.74–1.27)
2^nd ^year	12.2	13.8	9.8	11.3	
final	12.9	10.1	10.0	12.1	
Sore throat					
recruitment	10.4	13.7	10.2	11.4	1.14 (0.85–1.54)
2^nd ^year	11.6	11.7	11.1	7.8	
final	12.7	10.4	10.3	10.1	
Constant cough					
recruitment	6.0	7.9	7.0	6.3	1.11 (0.79–1.56)
2^nd ^year	6.4	7.2	8.1	6.2	
final	9.7	8.6	7.3	5.6	
Water retention					
recruitment	14.6	16.3	16.6	15.5	1.09 (0.79–1.50)
2^nd ^year	13.4	15.2	14.8	11.8	
final	16.2	13.6	16.1	15.1	
Loss of appetite					
recruitment	3.7	3.4	3.0	3.8	0.91 (0.69–1.20)
2^nd ^year	3.5	2.9	2.0	3.5	
final	3.0	3.4	3.3	3.3	
Vaginal bleeding					
recruitment	3.5	2.3	4.2	2.8	19.65 (12.15–31.79)
1^st ^year	10.3	2.7	9.5	1.2	
final	5.9	0.7	4.9	0.7	

* Among those whose data available

HT – hormone therapy

OR – combined odds ratio for treatment versus no-treatment arms

CI – confidence interval

**Figure 2:**
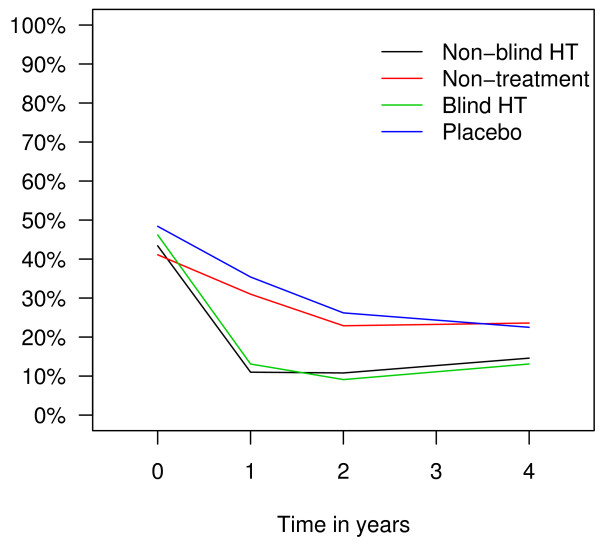
Prevalence of hot flushes in different trial arms in the EPHT Trial over time.

**Figure 3:**
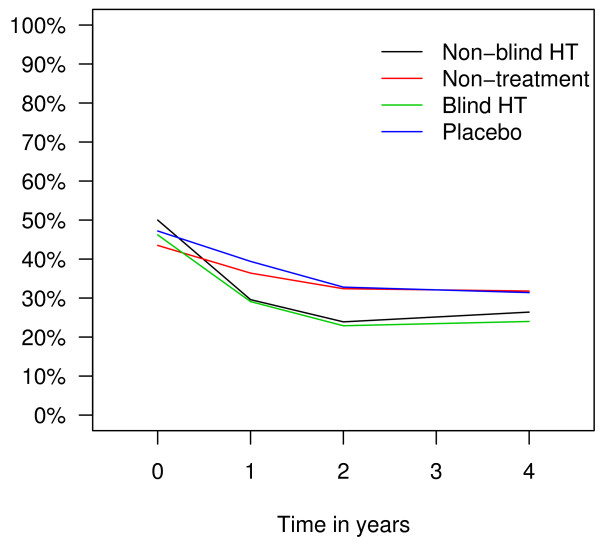
Prevalence of sweating in different trial arms in the EPHT Trial over time.

**Figure 4:**
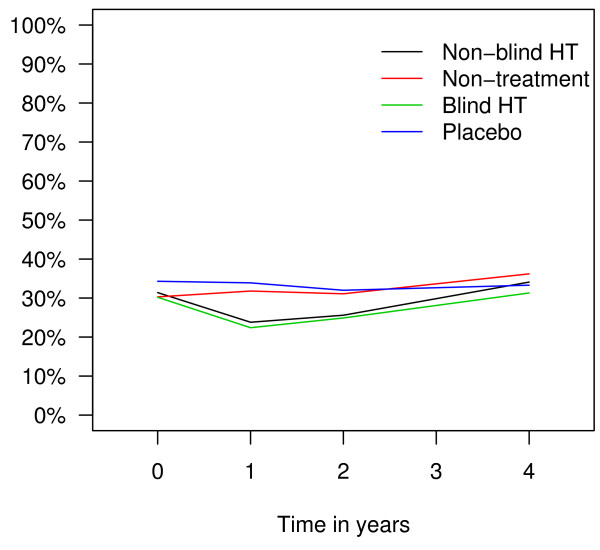
Prevalence of sleeping problems in different trial arms in the EPHT Trial over time.

Longitudinal data analysis showed that throughout the trial, less women reported hot flushes (OR 0.20; 95% CI: 0.14–0.28), sweating (OR 0.56; 95% CI: 0.44–0.72) and sleeping problems (OR 0.66; 95% CI: 0.52–0.84) in hormone therapy arms than in non-therapy arms. Prevalence of hot flushes in different trial arms over time is presented in Figure 2, prevalence of sweating in Figure 3, and prevalence of sleeping problems in Figure 4. The difference between the trial arms in reporting sleeping problems at the end of the trial was less evident than at the end of the second trial year probably due to decrease in adherence rates. There was no difference between the treatment and non-treatment arms in reporting depression or any other symptoms (Table 2).

Longitudinal data analysis showed that hormone therapy increased the risk of vaginal bleeding substantially (OR 19.65; 95% CI: 12.15–31.79). More women in hormone therapy arms than in non-therapy arms reported vaginal bleeding in the previous 12 months both at the end of the second trial year (58 versus 13) and at the end of the trial (41 versus 13). The mean number of bleeding episodes per woman reporting bleeding in the previous 12 months was also higher in hormone therapy arms both at the end of the second trial year (3.7 versus 2.1) and at the end of the trial (3.5 versus 1.2). Bleeding episodes were considered to have been heavy by more women in hormone therapy arms than in non-therapy arms at the end of the second trial year (3 versus 0) and at the end of the trial (4 versus 0).

At the end of the first trial year, less women in hormone therapy arms (5%) than in non-therapy arms (9%) reported painful intercourse (OR 0.59; 95% CI:0.37–0.94), but more women complained of painful intercourse in hormone therapy arms (6%) than in non-therapy arms (2%) at the end of the trial (OR 3.12; 95% CI:1.65–6.25). The proportion of women having had no intercourse was not significantly different in different trial arms over time.

According to the EQ-5D questions, there was no difference between the arms in reporting problems with self-care, usual activities, pain or discomfort, anxiety or depression, but problems with mobility were reported more often by women in the blind hormone therapy arm (35% of respondents) than by women in the placebo arm (27% of respondents) at the end of the trial (OR 1.43; 95% CI 1.01–2.05). Despite the differences in reporting symptoms and problems with mobility, there was no difference in the distribution of women with different EQ-5D scores in trial arms at the end of the second trial year and at the end of the trial. As the distribution of women with different quality of life scores was strongly skewed, the results are presented in quartiles (Table 3). For half of the women in all trial arms, EQ-5D score was 0.90 at the end of the second trial year and 0.80 at the end of the trial.

**Table 3:** Distribution of women with different quality of life scores in each arm of the EPHT Trial over time

	EQ-5D score				
Survey year/trial arm	Minimum	1^st ^quartile	Median	3^rd ^quartile	Maximum
2nd year					
Non-blind HT	0.30	0.80	0.90	1.00	1.00
Non-treatment	0.40	0.70	0.90	1.00	1.00
Blind HT	0.30	0.80	0.90	1.00	1.00
Blind placebo	0.30	0.80	0.90	1.00	1.00
Final					
Non-blind HT	0.40	0.70	0.80	0.90	1.00
Non-treatment	0.30	0.70	0.80	0.90	1.00
Blind HT	0.30	0.70	0.80	0.90	1.00
Blind placebo	0.10	0.70	0.80	0.90	1.00

EQ-5D – quality of life according to EQ-5D score

HT – hormone therapy

Longitudinal data analysis showed that quality of life decreased with age (estimated difference -0.027 per one decade, SE = 0.009) and time (estimated difference -0.017 per one trial year, SE = 0.0024), but was not influenced by treatment or blinding.

## Discussion

Our data showed a decrease in reporting vasomotor symptoms and sleeping problems in hormone therapy arms, and an increase in the rates of vaginal bleeding over the observed period of time. Despite the difference in symptom reporting, no difference in quality of life scores between different trial arms was observed.

Hormone therapy is not used only around menopause. Before the publication of the results from the Women's Health Initiative Trial and the Million Women Study, it was estimated to be used by a third of postmenopausal women in Sweden [24], by forty percent in the United States [25] and by more than forty percent in Finland [26] for up to fifteen years after menopause. After publication of the findings from these studies the use of hormone therapy declined [27-31], but still hormone therapy is used by tens of millions of women all over the world.

Women interested in participating in the trial were younger and more frequently reported depression and trouble sleeping than women not interested in participation. There was no difference in other socioeconomic characteristics and the reporting of other symptoms inquired about in the recruitment questionnaire [32].

We assume the higher proportion of women reporting sweats in the non-blind hormone therapy arm at recruitment to be a chance finding as treatment allocation was not known before recruitment. No significant difference in the numbers of women reporting sweats at baseline was observed between hormone therapy and non-treatment arms after combining data from both sub-trials. Therefore, the difference was not taken into account in the statistical analysis.

The proportion of women reporting problems in different trial arms at recruitment was higher than during the trial, probably because not all women with symptoms at recruitment responded to annual questionnaires. As the response rates to the annual questionnaires varied and the final questionnaire was completed at a different time, longitudinal data analysis was used to estimate the effect of hormone therapy on the prevalence of symptoms and quality of life over time.

No data for EQ-5D was collected at baseline. We assume that the EQ-5D scores at baseline were similar in all trial arms. Even though EQ-5D asks about mobility, self-care, usual activities, pain and anxiety or depression and is said to be one of the bluntest health-status instruments available [33], EQ-5D has been found to be suitable for measuring health-related quality of life among gynecological patients in all age groups [34]. It is more likely to elicit a response than complicated measurement tools [35].

The beneficial effect of hormone therapy on vasomotor symptoms is well established [4-7] as well as bleeding as its side-effect [11]. The effect of hormone therapy on sleep disturbance has earlier been reported over a shorter period of time [15,36]. Although transition to menopause has been found to be associated with depressed mood [37], we found no difference in the proportion of women reporting depression in different trial arms.

Low adherence rates in the treatment arms may have diluted the effect of hormone therapy. The inconsistent effect of hormone therapy on painful intercourse during the EPHT Trial could be explained by lower adherence rates at the end of the trial in comparison with the adherence rates in hormone therapy arms at the end of the first trial year. Women were not persuaded to continue trial treatment, and the fall in adherence rates was the same as in real life circumstances [38].

Overall quality of life scores decreased with increasing age. The problems with mobility reported more often by women in hormone therapy arms in the EQ-5D questionnaire at the end of the trial may be a chance finding. The results are presented separately for both the blind and the non-blind sub-trial. Blinding had no effect on symptom reporting or quality of life over time.

In the WHI Trial, moderate to severe hot flushes were reported by 24% of participants aged 50–54 years and 15% for women aged 55–59 years at baseline, and night sweats by 21% and 14% accordingly [14]. In the HERS Trial, hot flushes were reported by 16% of participants at baseline [13]. In the EPHT Trial, the proportion of women with vasomotor symptoms was higher (Table 2).

In the WHI Trial, changes in menopausal symptoms were analyzed at the end of the first trial year [14], and quality of life was measured at baseline, at year one, and in a sub-group of women in year three [15]. In the HERS Trial, quality of life and depressive symptoms were measured at a three-year follow-up [13]. In the EPHT Trial, data on symptom reporting was collected annually, data on quality of life at year two and at the end of the trial, the mean follow-up period being 3.6 years.

In the WHI Trial, quality of life was assessed with the use of the RAND-36 Health Survey, and in addition data about depressive symptoms, sleep disturbance, sexual and cognitive functioning, and menopausal symptoms were collected [15]. In the HERS Trial, physical activity was measured by the Duke Activity Status Index, energy and mental health by RAND scales, and depressive symptoms on the Burnam scale [13].

According to the WHI Trial data, combined hormone therapy improved vasomotor symptoms, vaginal or genital dryness, and all pain symptoms, but increased the rates of breast tenderness during the first year of use and vaginal bleeding persistently [14]. No clinically meaningful effect on health-related quality of life was detected [15]. The HERS trial data suggested that quality of life generally declined during the follow-up and hormone therapy had negative effects on physical function, but it improved depressive symptoms and quality of life for women with menopausal symptoms [13].

The follow-up period for symptom reporting and quality of life in the EPHT Trial was longer than in the WHI and HERS trials, while the regimen used was the same. The difference in outcomes can be explained by the difference in the participants' health indicators and age, by the difference in measurement tools, and by cross-cultural differences [39].

## Conclusion

Data from the EPHT Trial show that postmenopausal hormone therapy decreased hot flushes, sweating, and sleep disorders, but increased episodes of vaginal bleeding. No effect on quality of life was observed. Physicians prescribing hormone therapy for postmenopausal women should take into account the complex of the effects of hormone therapy.

Health-related quality of life may also be influenced by cultural and socio-economic factors [40], therefore all tools that measure quality of life are highly influenced by personality and social circumstances [41]. Which factors influence the quality of life in the ageing population in different cultural contexts deserves additional research, and measurement tools for quality of life could be further developed. The underlying mechanisms for subjective symptoms among postmenopausal women require future clinical research.

## Competing interests

The trial was supported by the Academy of Finland (grants 69838 and 201490), STAKES (National Research and Development Centre for Welfare and Health), Finland, the Estonian Science Foundation (grants 5203 and 6570) and the Estonian Ministry of Education and Research (target funding 0192112s02). Drugs were donated by Wyeth Ayerst Company via the Women's International Study of Long Duration Oestrogen After Menopause (WISDOM) in the United Kingdom.

The funding bodies had no role in the study design, data collection, data analysis, data interpretation, writing of the report or in the decision to submit the paper for publication.

## Authors' contributions

MR and EH contributed to the design of the trial, the development of questionnaires and to the manuscript; HK and SLH contributed to the development of questionnaires and to the manuscript; KF contributed to the data analysis and to the manuscript; PV contributed to the development of questionnaires, data collection and analysis, and to writing the manuscript.

## Pre-publication history

The pre-publication history for this paper can be accessed here:


